# Mapping and Candidate Gene Analysis of the Low-Temperature-Sensitive Albino Gene *OsLTSA8* in Rice Seedlings

**DOI:** 10.3390/cimb46070388

**Published:** 2024-06-27

**Authors:** Yu Wei, Xiaoqiong Li, Dongxiu Li, Xuejun Su, Yunchuan Huang, Qiuwen Li, Manling Liang, Xinghai Yang

**Affiliations:** 1 Guangxi Key Laboratory of Rice Genetics and Breeding, Rice Research Institute, Guangxi Academy of Agricultural Sciences, Nanning 530007, China; weiyu@gxaas.net (Y.W.); lixiaoqiong@gxaas.net (X.L.); 13557035653@163.com (D.L.); suxuejun@gxaas.net (X.S.); hyc19650563@163.com (Y.H.); liqiuwen051@163.com (Q.L.); lmanling@gxaas.net (M.L.); 2 State Key Laboratory for Conservation and Utillzation of Subtropical Agro-Bioresources, Nanning 530007, China

**Keywords:** rice, low temperature, albino, QTL-seq, RNA-seq, candidate genes

## Abstract

Chloroplasts are organelles responsible for photosynthesis in plants, providing energy for growth and development. However, the genetic regulatory mechanisms underlying early chloroplast development in rice remain incompletely understood. In this study, we identified a rice seedling thermosensitive chlorophyll-deficient mutant, *osltsa8*, and the genetic analysis of two F_2_ populations suggested that this trait may be controlled by more than one pair of alleles. Through reciprocal F_2_ populations and QTL-seq technology, *OsLTSA8* was mapped to the interval of 24,280,402–25,920,942 bp on rice chromosome 8, representing a novel albino gene in rice. Within the candidate gene region of *OsLTSA8*, there were 258 predicted genes, among which *LOC_Os08g39050*, *LOC_Os08g39130*, and *LOC_Os08g40870* encode pentatricopeptide repeat (PPR) proteins. RNA-seq identified 18 DEGs (differentially expressed genes) within the candidate interval, with *LOC_Os08g39420* showing homology to the pigment biosynthesis-related genes *Zm00001d017656* and *Sb01g000470*; *LOC_Os08g39430* and *LOC_Os08g39850* were implicated in chlorophyll precursor synthesis. RT-qPCR was employed to assess the expression levels of *LOC_Os08g39050*, *LOC_Os08g39130*, *LOC_Os08g40870*, *LOC_Os08g39420*, *LOC_Os08g39430*, and *LOC_Os08g39850* in the wild-type and mutant plants. Among them, the differences in the expression levels of *LOC_Os08g39050* and *LOC_Os08g39430* were the most significant. This study will contribute to further elucidating the molecular mechanisms of rice chloroplast development.

## 1. Introduction

Albinism is an aberrant phenomenon that takes place during the rice growth process, characterized by the whitening of leaf coloration. Due to the absence of chlorophyll in albino seedling leaves, photosynthesis cannot proceed normally [1], thereby impacting plant growth and development, and ultimately leading to a decrease in yield [2].

Researchers have elucidated the mechanisms and regulatory pathways underlying albinism in rice seedlings through genetic, physiological, and molecular approaches. Currently, several related genes have been cloned, including genes encoding chlorophyll synthesis enzymes, chlorophyll reductase, chlorophyll synthesis-related genes, and transcription factors. For instance, *RNRL1* affects rice leaf chlorophyll in a temperature-dependent manner: *RNRL1* mutant plants exhibit a white leaf phenotype under constant conditions of either 20 °C or 30 °C, while they are nearly fully green under alternating conditions of 30 °C and 20 °C [3]. The mutant *ysa* displays leaf whitening before the third leaf stage, with significantly lower contents of chlorophyll a, b, and carotenoids compared to the wild type. Subsequently, the leaves gradually turn green and completely recover to normal green coloration by the sixth leaf stage [4]. The *AM1* gene encodes a potassium ion efflux transporter, which plays a significant role in regulating the expression of genes related to chlorophyll biosynthesis, chloroplast development, and photosynthesis in the early leaves [5]. *tcd5* is a temperature-sensitive chlorophyll-deficient mutant, and the product encoded by the *TCD5* gene is a monooxygenase that affects chloroplast development under low-temperature conditions [6]. The *Albino Leaf1* variant exhibits a distinct albino phenotype with significantly reduced chlorophyll content. Further investigation revealed that this gene mediates the balance of photosynthesis, ribosomes, and metabolism in a dosage-dependent manner, thereby regulating chloroplast development [7]. The *SL2* gene encodes chloroplast 50S ribosomal protein L21, and the mutants display seedling albino lethality and almost undetectable levels of chlorophyll a, chlorophyll b, and carotenoids [8]. *AL2* encodes a chloroplast IIA intron splicing enhancer factor, which participates in the splicing of chloroplast IIA and IIB introns, coordinating the expression of a series of chloroplast-related genes and regulating rice chloroplast development [9]. The *OsARVL4* mutant exhibits changes in rice leaf veins and reverse curling, and is potentially regulated by epigenetic mechanisms such as DNA methylation that may modulate chlorophyll content and photosynthetic efficiency [10]. OsABCI8 is a conserved ATP-binding cassette transporter protein involved in the transport and homeostasis maintenance of transition metal elements such as iron. It participates in chloroplast development and chlorophyll synthesis, and is particularly crucial for chloroplast development under suboptimal light conditions [11,12]. *OsSLA4* may play a vital role in rice chloroplast early development and seedling growth by influencing the splicing of multiple chloroplast IIB introns [13]. The *ALM1* mutant displays albinism 1–2 days after seed germination, and its albinism phenotype becomes more and more significant as the plant grows. Chlorophyll a, b, and total chlorophyll content are significantly reduced in these mutants compared to the wild type [14]. *WAL3* is involved in chlorophyll synthesis- and photosynthesis-related metabolic pathways, with mutants exhibiting a reduced abundance of photosynthesis-related proteins [15]. *TSA* mutation causes the severe disruption of chloroplast ultrastructure, leading to significantly decreased levels of chlorophyll a, chlorophyll b, and carotenoids in rice leaf tissues [16]. *YLWS* encodes a P-type chloroplast-targeted PPR protein, participating in the splicing of chloroplast IIB introns and playing a crucial role in chloroplast development during early leaf development [17].

In previous studies, researchers used the QTL-seq method to map multiple albino phenotype genes in plants [18,19,20]. In this study, we constructed F_2_ populations using crosses between C6 × C13 and C13 × C6 rice lines. Extreme plants were selected from the two F_2_ populations for QTL-seq analysis. Through overlapping regions identified in both populations, the rice seedling albino gene was mapped to the interval of 24,280,402–25,920,942 bp on chromosome 8. RNA-seq analysis identified 18 differentially expressed genes within the candidate interval. This study lays the foundation for a deeper understanding of the chlorophyll metabolism during the rice seedling stage.

## 2. Results

### 2.1. Genetic Analysis

**Figure 1:**
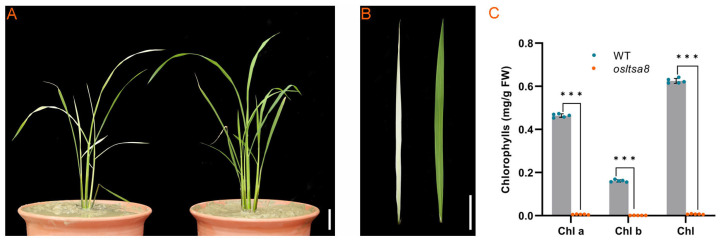
Phenotype of the low-temperature-sensitive albino mutant and wild type. (**A**) Plant. (**B**,**C**) Chlorophyll content in the wild-type and mutant (*osltsa8*) leaves. Student’s *t*-test was performed, *** *p* < 0.001. FD, fresh weight. Bar = 5 cm.

In the F_2_ populations of both C6 × C13 and C13 × C6 crosses, albino seedlings were observed when the temperature was below 20 °C (Figure 1A,B). In addition, the content of chlorophyll a and b in the mutants (*osltsa8*) was significantly lower than that in the wild-type (WT) plants (Figure 1C). The number of whitened leaves per plant ranged from two to five, typically exhibiting complete or mostly white leaf surfaces. Over time, the number of whitened leaves did not increase, and the whitened leaves gradually turned green. When the temperature exceeded 30 °C, whitened leaves showed a pattern where leaf veins turned green first, followed by the rest of the leaf gradually turning green, ultimately restoring to normal green coloration.

In the F_2_ population of ‘C6 × C13’, there were 64 albino seedlings and 386 normal seedlings. In the F_2_ population of ‘C13 × C6’, there were 62 albino seedlings and 628 normal seedlings. The ratios of albino seedlings to normal seedlings in the two populations were 6.03:1 and 10.1:1, respectively, deviating from the expected 3:1 ratio.

### 2.2. Whole-Genome Resequencing Analysis of Four Mixed Pools

A total of 50 normal leaf samples and 50 albino seedling samples were collected from the F_2_ population of ‘C6 × C13’, and equal amounts of DNA were extracted and mixed to form pools for normal leaves (A-14) and whitened leaves (B-14). Similarly, pools for normal leaves (A-22) and whitened leaves (B-22) were constructed using the F_2_ population of ‘C13 × C6’.

DNA from the two parents and the four pools were sequenced using the Illumina NovaSeq 6000 sequencing platform. After filtering, 26,986,277,446, 30,780,554,732, 35,147,706,412, and 30,665,949,474 clean bases were obtained from the four pools, respectively (Table S1).

Using the Nipponbare genome as a reference, we aligned the clean reads obtained from the sequencing to the reference genome using SAMtools version 1.9. The alignment rates of the two parents to the reference genome exceeded 98%, while the average alignment rates of the four mixed pools were 97.7%. The average sequencing depth of the two parents was 78×, and the average sequencing coverage of the four offspring pools was 78.5× (Table S2).

SNP detection was performed using a GATK (v3.8) HaplotypeCaller, resulting in 2,018,779 and 1,995,166 SNPs detected in the two parents after filtering. The four mixed pools yielded 1,516,204, 1,512,485, 1,517,079, and 1,517,627 SNPs, respectively (Table S3).

Before conducting the association analysis, we performed further filtering on the SNPs. A-14 vs. B-14 and A-22 vs. B-22 finally yielded 457,007 and 457,049 high-quality reliable SNPs, respectively.

### 2.3. Mapping of Genomic Regions Controlling Rice Seedling Albinism

#### 2.3.1. A-14 vs. B-14 Association Analysis

**Figure 2:**
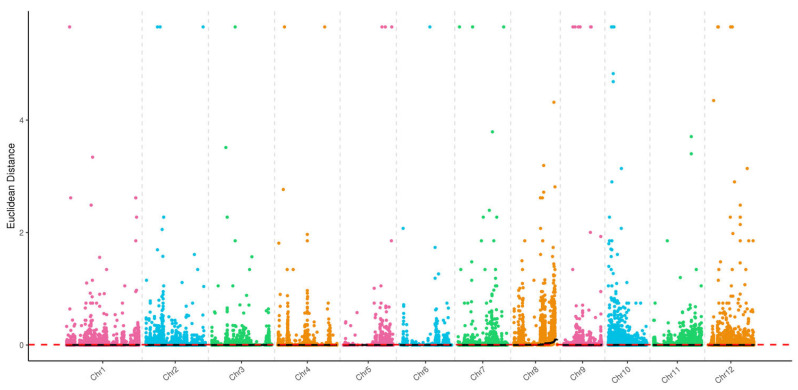
The distribution of ED-associated regions across 12 chromosomes in A-14 vs. B-14.

Using SNPs with genotype differences between the two mixed pools, the depth of each base in the different pools was calculated, and the ED (Extreme Difference) value was computed for each site. To eliminate background noise, the fifth power of the original ED values was used as the association value to effectively eliminate background noise. Then, the SNPNUM method was employed to fit the ED values, and the median plus 3 standard deviations (SD) of all fitted values were taken as the association threshold for analysis, resulting in a threshold value of 0.01. Based on this association threshold, two regions were identified on rice chromosome 8: 15,885,239–16,757,732 bp and 16,941,335–25,920,942 bp, with a total candidate genomic region length of 9.85 Mb (Figure 2).

**Figure 3:**
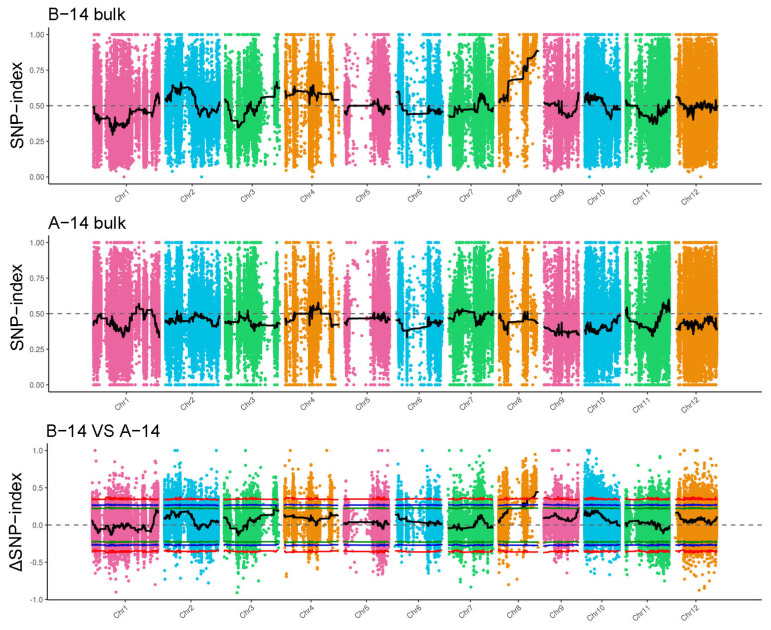
The distribution of SNP-index-associated regions across 12 chromosomes in A-14 VS B-14. The green line, blue line and red line represent the threshold of 90%, 95% and 99% confidence interval, respectively.

To eliminate false-positive sites, the ΔSNP-index values for markers on the same chromosome were fitted. In this study, we used the SNPNUM method to fit the ΔSNP-index values, and the regions with ΔSNP-index values above the association threshold were selected as regions associated with the trait. When the confidence level was set to 99%, the rice albino gene was mapped to the interval of 20,961,544–25,920,942 bp on rice chromosome 8 (Figure 3).

#### 2.3.2. A-22 vs. B-22 Association Analysis

**Figure 4:**
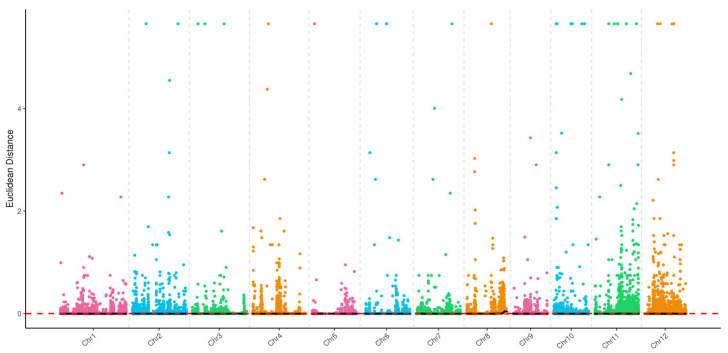
The distribution of ED-associated regions across 12 chromosomes in A-22 vs. B-22.

Using the ED method, we detected 23 candidate regions (≥10 kb) in the A-22 vs. B-22 comparison, distributed across all 12 rice chromosomes (Table S4). Among them, the largest candidate region was found on chromosome 8, spanning 7.92 Mb (Figure 4).

**Figure 5:**
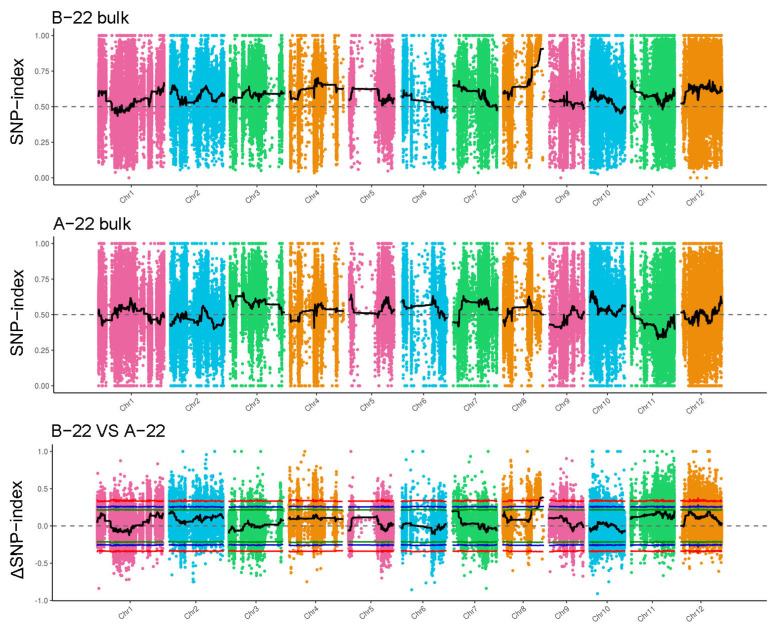
The distribution of SNP-index-associated regions across 12 chromosomes in A-22 vs. B-22. The green line, blue line and red line represent the threshold of 90%, 95% and 99% confidence interval, respectively.

With SNP-index method, we only detected one region associated with rice leaf variation on chromosome 8, spanning from 24,280,402 to 26,669,931 bp (Figure 5).

Therefore, we took the intersection of the candidate genomic regions identified in A-14 vs. B-14 and A-22 vs. B-22 comparisons, and the final determination of the candidate region for rice seedling albino gene was within the interval of 24,280,402 to 25,920,942 bp on chromosome 8. The gene was named *Low-Temperature-sensitive Albino Gene on Chromosome 8 in Oryza sativa* (*OsLTSA8*).

### 2.4. RNA-Seq Analysis

**Figure 6:**
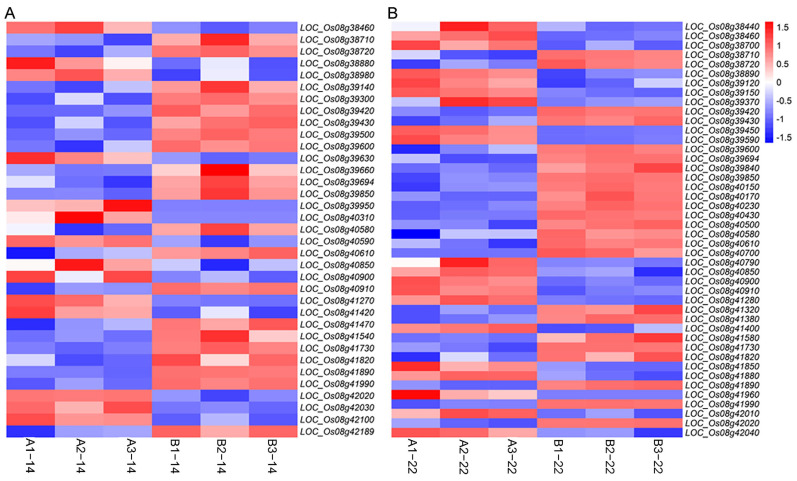
Differential expression genes between wild type and mutant. (**A**) ‘C6 × C13′ F_2_ population. (**B**) ‘C13 × C6’ F_2_ population. Boxes that are significantly differentially expressed are colored from −1.5 to 1.5.

The transcriptome sequencing data of 12 samples showed that each sample’s clean data exceeded 7.17 Gb and had a Q30 base percentage above 94.69% (Table S5). We then aligned the clean reads of each sample to the reference genome of *Oryza sativa* L. *japonica* (http://rice.uga.edu/, v_7.0, 20 July 2023), with alignment rates ranging from 92.55% to 93.29%. Gene expression levels were quantitatively analyzed using RSEM v 1.3.3 (http://deweylab.biostat.wisc.edu/rsem/, 20 July 2023), with FPKM as the quantification index. Next, we performed differential gene expression analysis between samples using DESeq2 v 1.24.0 (http://bioconductor.org/packages/stats/bioc/DESeq2/, 20 July 2023). The results showed that in ‘C6 × C13’, there were a total of 3916 differentially expressed genes (DEGs) between the wild type and the mutant (Table S6), with 35 DEGs located in the candidate genomic region (Figure 6A). In ‘C13 × C6’, there were 6033 DEGs between the wild type and the mutant (Table S7), with 44 DEGs located in the candidate genomic region (Figure 6B). There were 18 common DEGs between ‘C6 × C13’ and ‘C13 × C6’ (Table 1). Among these DEGs, the direct homologs of *LOC_Os08g39420*, *Zm00001d017656* (*GRMZM2G126517*), and *Sb01g000470* are involved in pigment biosynthesis (http://rice.uga.edu/, v_7.0, 20 July 2024, http://rice.uga.edu/cgi-bin/ORF_infopage.cgi?orf=LOC_Os08g39420, 20 July 2023). *LOC_Os08g39430* and *LOC_Os08g39850* are involved in the precursor synthesis of chlorophyll. Chloroplast precursors, also known as etioplasts, begin to turn green in the presence of light [21].

### 2.5. OsLTSA8 Candidate Gene Analysis

By using the Nipponbare sequence as the reference genome (http://rice.uga.edu/, v_7.0, 5 July 2023 ), we found 258 candidate genes within the 24,280,402 to 25,920,942 bp region of rice chromosome 8. Among them, *LOC_Os08g39050*, *LOC_Os08g39130*, and *LOC_Os08g40870* are annotated as pentatricopeptide proteins containing PPR domains (Pfam: PF01535.13). Plant PPR proteins are known to activate the expression of chloroplast genes [22] and participate in chlorophyll synthesis. However, the expression differences of these three genes in the RNA-seq analysis did not reach a significant level.

In the RNA-seq results, the expression levels of *LOC_Os08g39420*, *LOC_Os08g39430*, and *LOC_Os08g39850* were significantly upregulated in the wild type. Based on the analysis, we consider *LOC_Os08g39050*, *LOC_Os08g39130*, *LOC_Os08g40870*, *LOC_Os08g39420*, *LOC_Os08g39430*, and *LOC_Os08g39850* to be candidate genes for *OsLTSA8*.

*LOC_Os08g39840* and *LOC_Os08g39850* are annotated as lipoxygenases, and are chloroplast precursors. *LOC_Os08g39840* is located in the chloroplast [23] and participates in jasmonic acid biosynthesis, enhancing rice resistance to brown planthoppers [24].

### 2.6. RT-qPCR Validation

**Figure 7:**
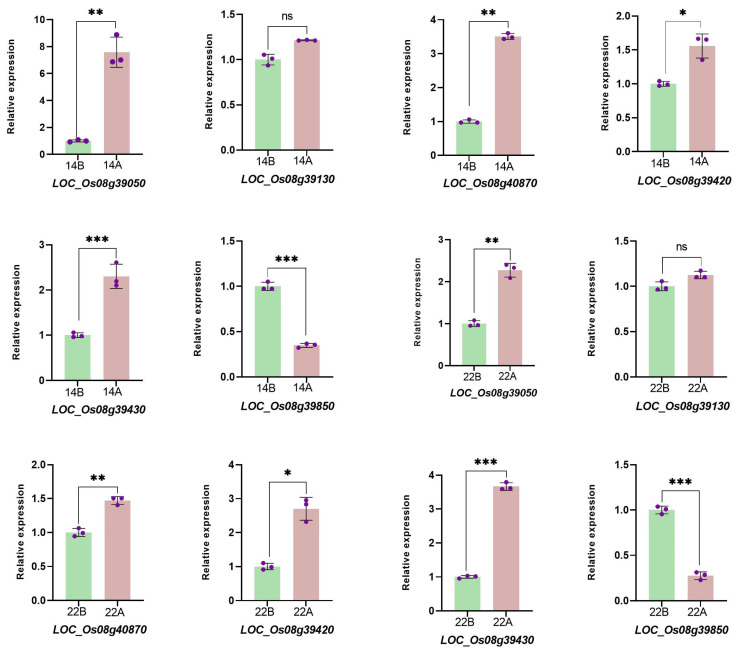
Using RT-PCR to analyze the expression levels of candidate genes. Student’s *t*-test was performed, ns means Non-Significant, * *p* < 0.1, ** *p* < 0.01, *** *p *< 0.001.

Based on the analysis of candidate genes, we selected *LOC_Os08g39050*, *LOC_Os08g39130*, *LOC_Os08g40870*, *LOC_Os08g39420*, *LOC_Os08g39430,* and *LOC_Os08g39850* for qPCR validation. The results showed that the expression levels of *LOC_Os08g39050*, *LOC_Os08g40870*, *LOC_Os08g39420*, and *LOC_Os08g39430* were significantly upregulated in the wild type. However, *LOC_Os08g39850* was significantly downregulated in the wild type (Figure 7).

## 3. Discussion

When rice seedlings encounter low temperature stress, chlorophyll synthesis and other processes are significantly inhibited, resulting in albino seedlings. At present, many genes related to low-temperature albino seedlings in rice have been identified and cloned [6,16,25,26,27,28,29,30], preliminarily elucidating the molecular regulatory network of chlorophyll synthesis and chloroplast development in rice under low-temperature conditions. However, the low-temperature chlorophyll synthesis and chloroplast development processes in rice are very complex, and there is an urgent need to identify and clone new genes. In this study, we identified a rice low-temperature-sensitive albino gene, *OsLTSA8*, which manifests albino symptoms under low temperatures but can recover normal growth when temperatures rise, consistent with previous research results [25,27,28]. Using QTL-seq, we mapped *OsLTSA8* within a 1.64 Mb region on rice chromosome 8. To date, no genes related to rice albino or yellowing have been reported in this region. Therefore, *OsLTSA8* represents a novel rice gene responsible for the low-temperature-sensitive albino phenotype in seedlings.

Based on gene annotation information, RNA-seq, and RT-qPCR analyses, we identified six candidate genes for *OsLTSA8*, among which *LOC_Os08g39050*, *LOC_Os08g39130*, and *LOC_Os08g40870* are associated with PPR proteins. PPR proteins constitute a large class of sequence-specific RNA-binding proteins encoded by the nucleus, characterized by the presence of multiple PPR motifs. Studies have shown that nuclear-encoded PPR proteins can activate the expression of chloroplast genes, participate in regulating the RNA splicing of rice chloroplast genes, and influence leaf color [13,28,31,32,33]. In studies of rice albino or yellowing phenotypes, several genes, including *YLWS* [17], *TCD10* [27], *CDE4* [28], *YSA* [4], *OsSLA4* [13], *OsPPR11* [34], *WLS4* [31], *OsPPR6* [32], *OsSLC1* [33], and *SSA1* [35], all encode PPR proteins. *YLWS* encodes a novel P-type chloroplast-targeted PPR protein with 11 PPR motifs. Further expression analysis indicates significant changes in genes encoded by many nuclear and plastid genomes at both the RNA and protein levels in *ylws* mutants. Chloroplast ribosome biogenesis and chloroplast development are impaired in *ylws* mutants under low-temperature conditions [17]. *TCD10* encodes a novel PPR protein primarily localized in chloroplasts, consisting of 27 PPR motifs. tcd10 mutants exhibit an albino phenotype with chloroplast deformities and fail to survive beyond the five-leaf stage when grown at 20 °C, but they display a normal phenotype at 32 °C [27]. *CDE4* encodes a P-type PPR protein located in chloroplasts, directly binding to transcripts of chloroplast genes rpl2, ndhA, and ndhB. The intron splicing of these transcripts is defective in cde4 mutants at 20 °C but normal at 32 °C [28]. The candidate genes *LOC_Os08g39050*, *LOC_Os08g39130*, and *LOC_Os08g40870* contain 7, 11, and 4 PPR motifs, respectively. Currently, the functions of the PPR proteins encoded by these three genes have not been explored. Therefore, a deeper analysis of *OsLTSA8* function may help elucidate the roles of novel PPR protein genes in rice growth and development.

In plant pigments, chlorophyll interacts with carotenoids, flavonoids, and betalains to give plants a vibrant array of colors [36,37]. In this study, the candidate gene *LOC_Os08g39420* may be involved in pigment biosynthesis, with its maize homolog genes *Zm00001d017656* and *Sb01g000470* primarily participating in indigoidine synthase (PTHR10584:SF201, PF04227). Indigoidine is a deep blue natural pigment, and its association with chlorophyll has not been observed so far. Chloroplast precursors, also known as etioplasts, can transform into chlorophyll upon exposure to light [21]. *LOC_Os08g39430* and *LOC_Os08g39850* are involved in chlorophyll precursor synthesis. *LOC_Os08g39430* encodes thylakoid lumenal protein. The thylakoid membrane inside chloroplasts is the main site for plant pigment metabolism, and changes in its internal structure are closely related to leaf color abnormalities. Temperature stress typically leads to the disruption of the overall integrity of the chloroplast thylakoid membrane, especially its damage, ultimately hindering chloroplast development [38].

This study identified a new low-temperature-sensitive albino gene *OsLTSA8* in rice seedlings, which was mapped in the 1.64 Mb interval on chromosome 8. Using the Rice Genome Annotation Project database, it was predicted that this region contains 258 genes, among which *LOC-Os08g39050*, *LOC-Os08g39130*, and *LOC-Os08g40870* encode PPR proteins. RNA seq and RT qPCR revealed significant differences in expression levels between the wild-type and mutant plants of *LOC-Os08g39050* and *LOC-Os08g39430*. This study further enriches the regulatory network of chloroplast development in rice under low temperatures, laying the foundation for cloning the *OsLTSA8* gene.

## 4. Materials and Methods

### 4.1. Plant Materials

C6 is a maintainer line of *indica* rice, which is bred by crossbreeding Tianzhe B11-2 as the female parent and Jinye B2-9 as the male parent, and then continuously self-crossing for 7 generations. C13 is also a maintainer line of *indica* rice, using Taifeng B as the female parent and Funong B as the male parent, and it is bred through 11 consecutive generations of self-crossing. The seedlings of C6 and C13 planted in Nanning have normal green leaves. We constructed two F_2_ populations by crossing C6 with C13 and crossing C13 with C6. Rice materials were planted in Nanning, Guangxi, in March 2023 under normal field management. The average temperature in Nanning in March 2023 was 23 °C.

### 4.2. Determination of Chlorophyll Content

We collected the leaves of wild-type and mutant plants from fresh F2 populations of C6 crossed with C13. The leaves were washed, dried, and cut into small pieces. After mixing, we weighed out 0.2 g samples and placed them in a mortar. A small amount of liquid nitrogen was added to grind them into powder. Then, 2 mL of 95% ethanol was added to the mortar to grind them into a homogenate, and 5 mL of ethanol was added and grinding continued until the tissue turned white. Filter into a 25 mL brown volumetric flask, rinse the mortar, grinding rod, and residue several times with a small amount of ethanol, and finally filter together with the residue into the volumetric flask. Use a dropper to absorb the ethanol and wash all the chloroplast pigments on the filter paper into a volumetric flask until there is no green color on the filter paper or residue. Finally, dilute to 25 mL with 95% ethanol and shake well. Pour the chloroplast pigment extraction solution into a colorimetric dish with a light path of 1 cm. Using 95% ethanol as the blank, measure the absorbance using a UV–visible spectrophotometer at wavelengths of 665 nm, 649 nm, and 470 nm.

### 4.3. DNA Extraction and Pooling

In each of the two F_2_ populations, 50 individuals with normal leaves and 50 individuals with whitened leaves were selected. DNA extraction was performed using the CTAB method. DNA concentration was measured using a Qubit 4 Fluorometer (Invitrogen, California, USA) to ensure concentrations greater than 2 ng/μL, and DNA purity and integrity were assessed via agarose gel electrophoresis. Equal amounts of DNA from 50 offsprings were pooled to form a mixed pool for each population.

### 4.4. DNA Sequencing and Quality Control

The sequencing of two parents and four mixed pools was performed using the Illumina NovaSeq 6000 sequencing platform. High-throughput sequencing generated raw data files from the original images, which were then converted into raw sequencing reads through Base Calling analysis. To ensure the quality of the subsequent information analysis, the raw reads were filtered to obtain clean reads. The main steps of data filtering were as follows:

(i) Removal of reads containing adapters;
(ii) Filtering out reads with a nucleotide (N) content exceeding 10%;
(iii) Removal of reads with more than 50% of bases with a quality score below 10.

### 4.5. Alignment to the Reference Genome

The Nipponbare reference genome (http://rice.uga.edu/, v_7.0, 5 July 2023 ) was used for alignment. SAMtools (V 1.9.) flagstat/depth commands (5 July 2023) were employed to calculate alignment rate, efficiency, sequencing depth, and coverage information for the samples.

### 4.6. SNP Detection

The detection of SNPs (single-nucleotide polymorphisms) was mainly achieved using GATK v3.8 [39] (https://software.broadinstitute.org/gatk/download/, 20 July 2023). Based on the localization results of the clean reads in the reference genome, we used SAMtools V 1.9 (https://github.com/samtools/samtools.git, 20 July 2023) to filter out redundant reads to ensure the accuracy of the detection results. Then, the HaplotypeCaller (local monotypic assembly) algorithm of GATK was used for SNP mutation detection. Each sample first generates its own gVCF and then performs population join genotyping. Finally, after strict filtering, the final set of mutation sites is obtained, which is usually stored in a VCF file.

The main filtering parameters were as follows:

(i) Based on the subroutine vcfutils.pl (varFilter w 5-W 10) in bcftools, SNPs within 5 bp near InDel (insertion–deletion) were analyzed.
(ii) ClusterSize 2 clusterWindow size 5 indicates that the number of mutations within a 5 bp window should not exceed 2.
(iii) QUAL < 30, a quality value in phred format, indicates the possibility of variant variation at this locus. Filter out those with a quality value below 30.
(iv) QD (quality by depth) < 2.0: The ratio obtained by dividing the quality of variation by the depth of coverage is the sum of the coverage depths of all samples containing mutated bases at this locus. Filter out those with QD below 2.0.
(v) MQ (mapping quality) < 40: The root mean square of the comparison quality values for all reads aligned to that site. Filter out MQ below 40.
(vi) FS > 60.0. The *p*-value conversion obtained through Fisher’s test describes whether there is significant positive or negative chain specificity for reads that only contain mutations and reads that only contain reference sequence bases during sequencing or alignment. That is to say, there will be no chain-specific alignment results, and FS should be close to zero. Filter out those with FS higher than 60.

Based on the position of the variant sites in the reference genome and the gene position information on the reference genome, the regions containing the variant sites (intergenic regions, genic regions, CDS regions, etc.) were determined. SnpEff v4.3 [40] (https://pcingola.github.io/SnpEff/, 5 July 2023) was utilized to predict the effects of the variations.

### 4.7. High-Quality SNP Filtering

Before the association analysis, SNPs were filtered based on the following criteria:

(i) SNPs with multiple genotypes were filtered out, retaining only biallelic genotype sites;
(ii) SNPs with read support less than 4 in the mixed pools were filtered out;
(iii) SNPs where the genotypes were homozygous and consistent among the mixed pools were filtered out;
(iv) SNPs where the genotypes were homozygous and consistent between the two parents were filtered out;
(v) For the recessive mixed pool, SNPs where the genotype did not originate from the recessive parent were filtered out; for the dominant mixed pool, SNPs where the genotype did not originate from the dominant parent were filtered out.

### 4.8. Euclidean Distance Method

The ED algorithm [41] is utilized to identify significant differential markers between mixed pools using sequencing data and to assess the association with trait-related regions. Theoretically, apart from the differential sites related to the target trait, the other sites between the two mixed pools constructed in the BSA project tend to be consistent. Therefore, the ED value of non-target sites should tend towards 0.

### 4.9. SNP-Index Method

The SNP-index [42] is a method published in recent years for performing marker association analysis based on the difference in genotype frequency between mixed pools. It primarily aims to identify significant differences in genotype frequency between mixed pools, as quantified by Δ (the SNP-index). The stronger the association between the marker SNP and the trait, the closer Δ (the SNP-index) is to 1.

### 4.10. RNA Extraction, Library Construction, and RNA Sequencing

During the four-leaf stage of rice, the F_2_ population was treated at 20 degrees Celsius. On the 7th day, when albino seedlings appeared, samples were taken and quickly frozen in liquid nitrogen before being transferred to a −80 °C freezer for storage. We grouped 50 green leaf plants and 50 albino leaf plants from the ‘C6 × C13’ F_2_ population into three sets, and extracted RNA from each set. These were then pooled equally to form three biological replicates. Similarly, we treated 50 green leaf plants and 50 albino leaf plants from the ‘C13 × C6’ F_2_ population in the same manner, resulting in three biological replicates. Subsequently, we conducted transcriptomic sequencing on these 12 samples. The procedures of RNA extraction and the detection of concentration, purity, and RNA integrity were completed as described in a previous study [43]. The quality control methods for RNA sequencing data, sequence alignment analysis (reference genome: http://rice.uga.edu/, 20 July 2024), gene function annotation, and expression difference analysis are referenced in previous research [44]. Fragments per kilobases per million reads (FPKM) were used to standardize gene expression.

### 4.11. RT-qPCR Analysis

We performed RT qPCR experiments using the RNA extracted in step 4.10. *OsActin1* was used as the reference gene [1]. RT-qPCR analysis was performed as described in a previous study [44]. qPCR primers are listed in Table S8.

### 4.12. Statistical Analysis

Excel 2019 and Graphpad Prism 10 were used for data statistics. CorelDRAW X8 (v18.1.0.661) was used for drawing. Primers were designed using Primer 5 software.

## Figures and Tables

**Table 1:** Overlapping differentially expressed genes between two populations within the candidate interval.

Gene	Functional Annotation
LOC_Os08g38460	zinc finger, C3HC4-type domain containing protein, expressed
LOC_Os08g38710	uncharacterized glycosyltransferase, putative, expressed
LOC_Os08g38720	cytochrome c oxidase assembly protein COX15, putative, expressed
LOC_Os08g39420	uncharacterized protein yeiN, putative, expressed
LOC_Os08g39430	thylakoid lumenal 19 kDa protein, chloroplast precursor, putative, expressed
LOC_Os08g39600	RNA methyltransferase, TrmH family protein, putative, expressed
LOC_Os08g39694	cytochrome P450, putative, expressed
LOC_Os08g39850	lipoxygenase, chloroplast precursor, putative, expressed
LOC_Os08g40580	methyltransferase domain containing protein, expressed
LOC_Os08g40610	30S ribosomal protein S16, putative, expressed
LOC_Os08g40850	mitochondrial carrier protein, putative, expressed
LOC_Os08g40900	auxin response factor, putative, expressed
LOC_Os08g40910	expressed protein
LOC_Os08g41730	peptidase, T1 family, putative, expressed
LOC_Os08g41820	exo70 exocyst complex subunit domain containing protein, expressed
LOC_Os08g41890	microtubule-associated protein, putative, expressed
LOC_Os08g41990	aminotransferase, putative, expressed
LOC_Os08g42020	zinc ion binding protein, putative, expressed

## Data Availability

The QTL-seq and RNA-seq data that support the findings of this study have been deposited at the National Center for Biotechnology Information (NCBI) Sequence Read Archive (SRA) with the accession codes PRJNA1125040 (SRA no. from SRR29442753 to SRR29442758) and PRJNA1125039 (SRA no. from SRR29442741 to SRR29442752).

## Supplementary Materials

The following supporting information can be downloaded at: https://www.mdpi.com/article/10.3390/cimb46070388/s1, Table S1: Two parents and four DNA pools resequencing data.; Table S2: The clean reads of two parents and four DNA pools mapping to reference genome; Table S3: High quality SNPs of two parents and four DNA pools; Table S4: Candidate genome region of A-22 vs B-22 pools using ED method; Table S5: Statistics of transcriptome sequencing data; Table S6: Differentially expressed genes of A-14 VS B-14; Table S7: Differentially expressed genes of A-22 VS B-22; Table S8: Primers for qPCR analysis.
